# Understanding ‘saturation’ of radar signals over forests

**DOI:** 10.1038/s41598-017-03469-3

**Published:** 2017-06-14

**Authors:** Neha Joshi, Edward T. A. Mitchard, Matthew Brolly, Johannes Schumacher, Alfredo Fernández-Landa, Vivian Kvist Johannsen, Miguel Marchamalo, Rasmus Fensholt

**Affiliations:** 1 Department of Geosciences and Natural Resource Management, University of Copenhagen, Copenhagen, Denmark; 2 School of GeoSciences, University of Edinburgh, Edinburgh, EH9 3FF United Kingdom; 3 School of Environment and Technology, University of Brighton, Cockcroft Building, Lewes Road, Brighton, BN2 4GJ UK; 4 AGRESTA Sociedad Cooperativa, C/ Numancia 1, Soria, E-42001 Soria Spain; 5 Departamento de Ingeniería y Morfología del Terreno, Universidad Politécnica de Madrid, E-28040 Madrid, Spain

## Abstract

There is an urgent need to quantify anthropogenic influence on forest carbon stocks. Using satellite-based radar imagery for such purposes has been challenged by the apparent loss of signal sensitivity to changes in forest aboveground volume (AGV) above a certain ‘saturation’ point. The causes of saturation are debated and often inadequately addressed, posing a major limitation to mapping AGV with the latest radar satellites. Using ground- and lidar-measurements across La Rioja province (Spain) and Denmark, we investigate how various properties of forest structure (average stem height, size and number density; proportion of canopy and understory cover) simultaneously influence radar backscatter. It is found that increases in backscatter due to changes in some properties (e.g. increasing stem sizes) are often compensated by equal magnitude decreases caused by other properties (e.g. decreasing stem numbers and increasing heights), contributing to the apparent saturation of the AGV-backscatter trend. Thus, knowledge of the impact of management practices and disturbances on forest structure may allow the use of radar imagery for forest biomass estimates beyond commonly reported saturation points.

## Introduction

Forests are vital regulators of global climate and an important source of livelihood for hundreds of millions of people^1, 2^. The increasing anthropogenic threat to forests^3, 4^ has led to a surge in research aimed at accurately mapping forest carbon, largely in support of global efforts on Reducing Emissions from Deforestation and forest Degradation (REDD+)^5, 6^ and achieving sustainable forestry^7^. Such research has shown that satellite radar images are reliable indicators of forest aboveground biomass (AGB) up to a point where sensitivity to AGB is lost. This has enabled them to be widely used in quantifying standing carbon stocks and changes^8–13^. Moreover, interest has grown in using radar backscatter to map subtle dynamics of forest changes, specifically including forest degradation and regrowth^9, 14–16^. The results are robust and promising, such that data from a number of recent (e.g. Sentinel-1, Advanced Land Observing Satellite-2 (ALOS-2)) and future (e.g. BIOMASS, Satellites for Observation and Communications (SAOCOM), NASA-ISRO Synthetic Aperture Radar (NISAR)) satellite radar missions will be used to this end. The longest, and hence most sensitive^17^, space-borne radar wavelength currently available for forest monitoring is ~23 cm L-band used by the ALOS-2 satellite (preceded by ALOS in 2006–2011).

However, the fundamental fact that backscatter is not a direct measure of forest biomass, but is governed by vegetation structural properties that may be related to it, poses a challenge in the science of mapping AGB with radar^18^. Microwave energy transmitted by Synthetic Aperture Radar (SAR) systems is primarily scattered by vegetation components comparable in size to the wavelengths used (e.g. stems and branches, which also contain the majority of forest biomass, for L-band and P-band waves). Hence, it is expected from theory that the physical properties of these components (e.g. stem sizes and number densities) will influence the AGB-backscatter relationship^17, 19^ and signal ‘saturation’ (i.e. the decreased sensitivity of backscatter to AGB above a certain AGB value). Extensive effort has been directed to modelling vegetation-backscatter interaction, e.g. through the seminal Water Cloud^20^ or ‘random volume over ground’ models^21^, which assume a constant extinction of microwave energy as a function of canopy depth and are extended to numerous other radiative transfer models^22–24^. However, there is still a relative lack of studies linking such models to the range of macroecological growth patterns (e.g. changing stem sizes and tree number densities as forests age) observed across the world’s forests. In the context of predicting AGB, this is important for two reasons. First, theoretical models have shown that saturation may not solely be caused by increasing canopy opacity to radar waves as AGB increases, but also by variations in these macroecological structural properties^25–27^. Only models incorporating structural information can explain why saturation rates differ across forest types and why it is observed even in sparse canopy forests (e.g. open woodlands^28^). Dependency on structure can also explain the counter-intuitive decrease in backscatter as forests transition from high to very high biomass ranges (e.g. 200 Mg/ha to 500 Mg/ha)^29, 30^ beyond saturation points. However, only a few empirical studies have discussed, validated or incorporated such results when mapping AGB^19, 28, 31, 32^, mostly due to the lack of large-scale measurements of forest structure. With over 35 % of the world’s forested area estimated to contain AGB beyond the commonly reported saturation point for L-band SAR (~100 Mg/ha) (estimated from Avitabile *et al*.^33^ and GEOCARBON^34^), this knowledge gap is a major limitation to the use of the latest SAR satellite data for global forest monitoring. Second, as structural composition varies greatly both due to natural resource competition and stand-level management practices, it can be expected that even forests containing the same tree types (e.g. even-aged monospecific vs. semi-natural unmanaged conifer plantations) will show differing AGB-backscatter relationships. Theoretical models of Woodhouse^25^ and Brolly & Woodhouse^26^ show that a strong relation of AGB with backscatter can be expected when age-related structural development (e.g. increasing stem sizes and number densities) is correlated with increasing AGB. However, forests that do not follow such development (e.g. increasing stem sizes, but decreasing number density due to natural and anthropogenic thinning, with increasing AGB) may confound our interpretation of the AGB-backscatter relationship^18^. Although often acknowledged, these critical aspects risk being unaccounted for in empirical studies. This is especially crucial in studies that relate backscatter to ground-measurements of AGB, or aboveground stand volume (AGV, from which AGB is derived by multiplying with a constant wood-density value^35^), across a variety of forest types and structural gradients^36–38^. If backscatter is determined by physical forest characteristics, these aspects are vital to increase our understanding of what exactly is being quantified when forest stand volume, biomass or changes therein, are mapped with SAR images.

**Figure 1:**
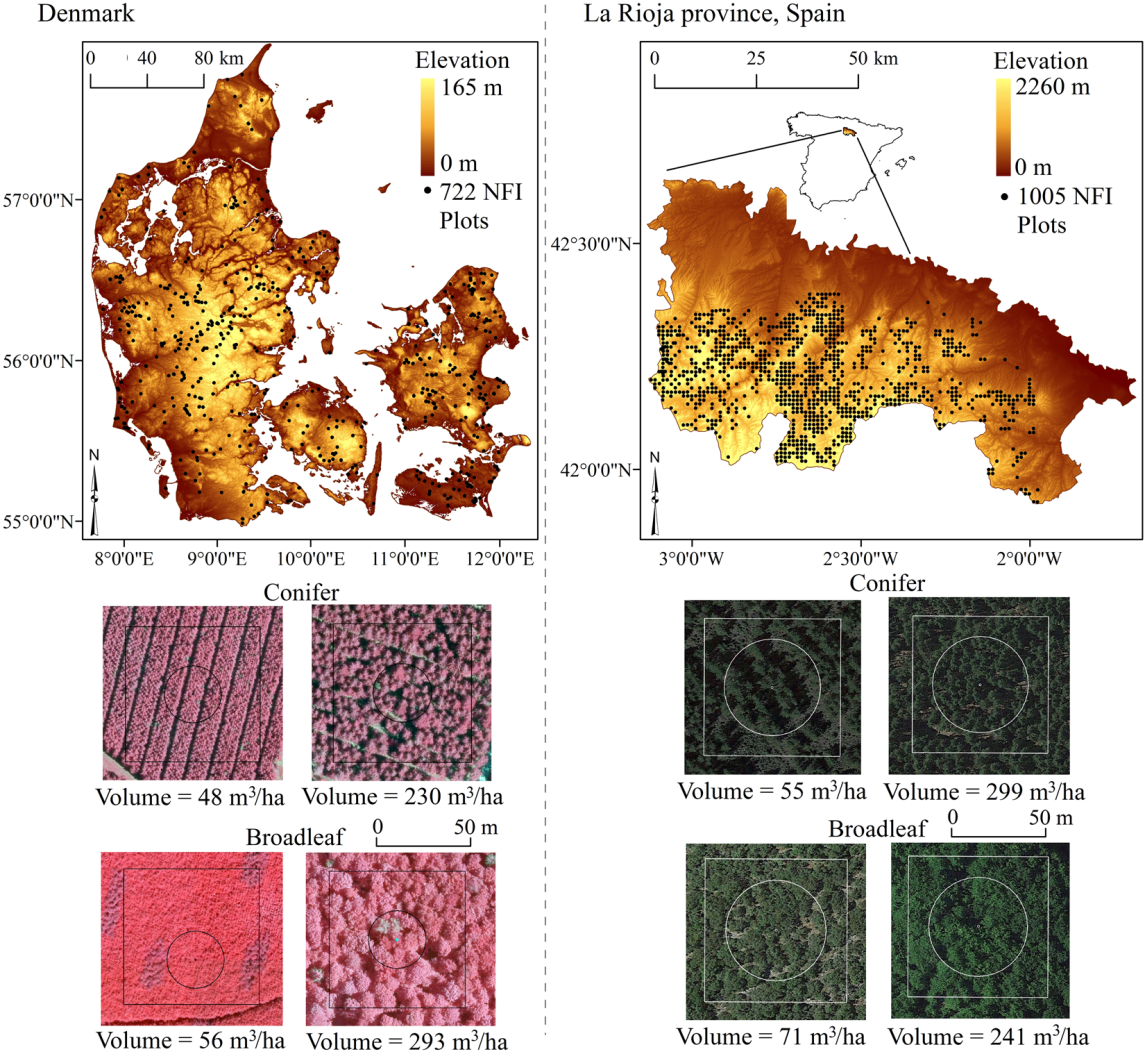
National Forest Inventory (NFI) plots in Denmark and La Rioja, Spain. Plot locations and examples of aerial photographs of selected conifer and broadleaf forests in each study site are provided. On the aerial photographs, circles represent NFI plots and squares represent the larger square plots (71 m × 71 m size) from which lidar metrics and SAR backscatter were extracted. Lidar metrics extracted from the NFI and square plots are compared in Supplementary Figure S8. The map was produced using software ArcGIS 10.1 (http://www.esri.com/software/arcgis).

**Figure 2:**
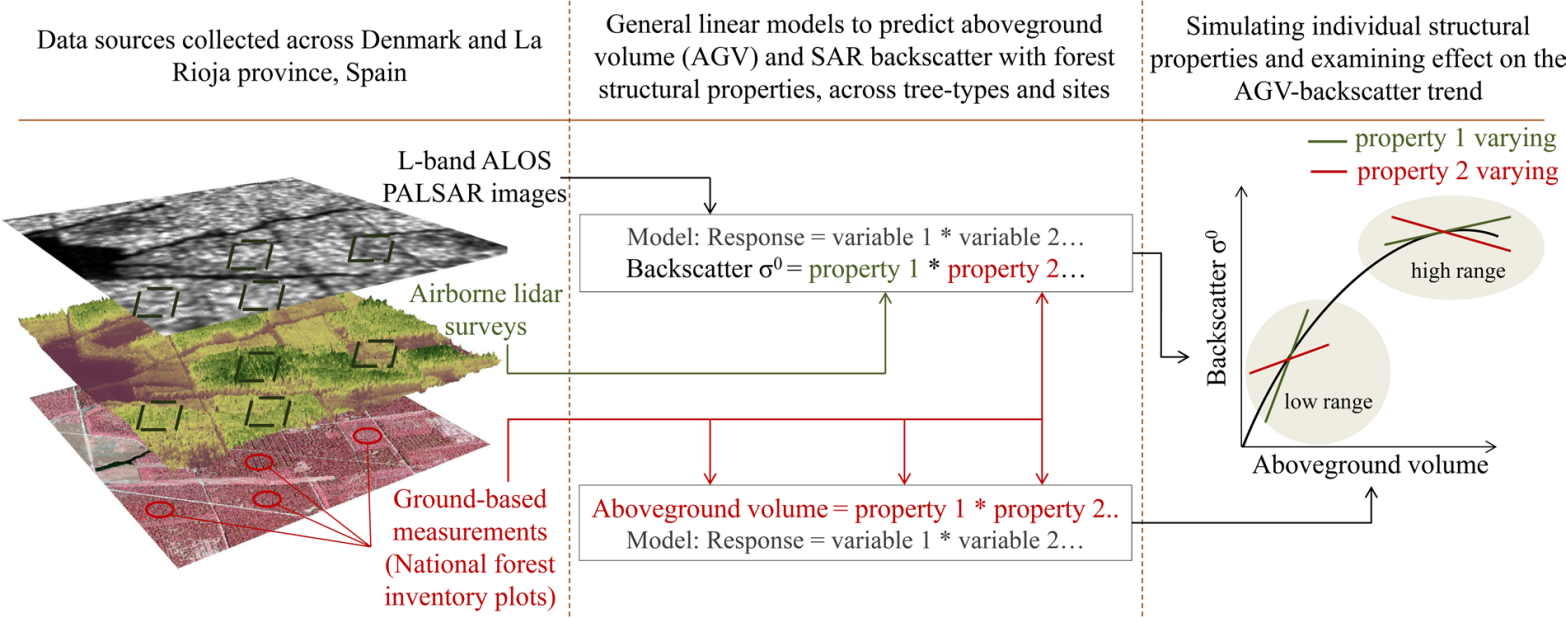
Schematic diagram of data sources and methodology used in this study. General linear models were used to predict SAR backscatter and forest aboveground volume (AGV). Each predictor variable was then varied individually to test its influence on the slope of the AGV-backscatter trend. The maps were produced using software ArcGIS 10.1 (http://www.esri.com/software/arcgis).

This study deconstructs the AGV and L-band SAR backscatter relation using empirical statistical models, and compares the trends exhibited in the relation in forests with varying structures. The study is conducted in forests with two common temperate tree types - broadleaves and conifers - across a unique set of 1727 plots in La Rioja province, Spain, and Denmark (Fig. 1). The forests were subjected to different management regimes and were measured with wall-to-wall airborne lidar surveys and on-ground National Forest Inventories (NFIs) over 5 years. Our approach involves two steps, including (1) simultaneously relating various forest structural properties to backscatter using statistical models and (2) simulating forests in two common ranges (low AGV and high AGV) to test the influence of each property on backscatter individually at these ranges (Fig. 2). We identify (1) which physical properties of stems and stand-level structure influence the AGV-backscatter relationship, and (2) if, and how, variations in each of these physical properties can explain the saturation of backscatter with increasing AGV. These objectives are achieved by analysing the trends in the relations and the statistical significance of various predictor variables in empirical models, rather than comparing absolute values, across the two study sites.

We find that accounting for differences in structures of forests with different tree-types, and in the growth trajectories of structural properties, can explain significant differences observed in AGV-backscatter trends in the two independent sites. Common age-related structural changes in forests simultaneously increase and decrease backscatter, providing an explanation for its apparent ‘saturation’. This challenges the widely held view that only increased vertical canopy opacity drives saturation, and could allow mapping AGV beyond commonly stated saturation points. We discuss the insights that this study provides into the requirements for accurate large-scale AGV mapping, and detecting deforestation and forest degradation, using SAR.

## Results

### Forest structural properties describing backscatter and AGV

**Figure 3:**
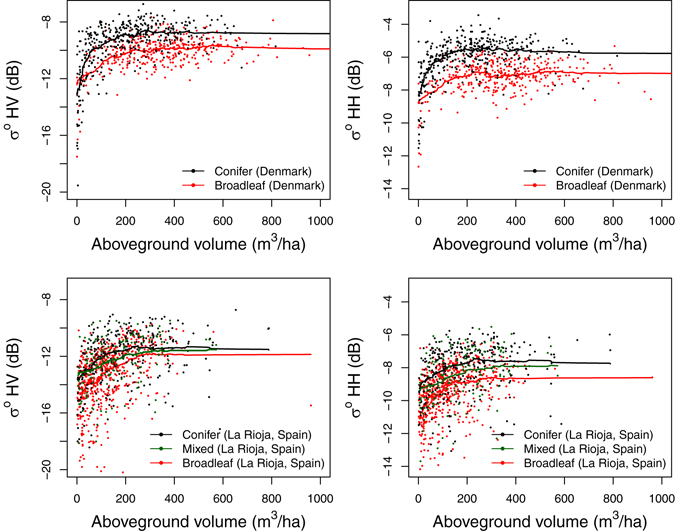
Distribution of radar backscatter and aboveground volume (AGV) in forests of different tree-types. Solid lines show a smoothed moving average trend of 50 and 100 observations in Denmark and La Rioja respectively (ignoring missing values). General linear models (GLMs) show statistically significant differences (*p* < 0.001) in backscatter between broadleaf and conifer tree-types in both sites.

A number of general linear models (GLMs) were built to test the relationship between (1) backscatter and AGV, and (2) backscatter and other variables describing forest structure. In both Denmark and La Rioja, a strong correlation between backscatter (polarizations horizontal-transmit and vertical-receive, ${\sigma }_{{\rm{HV}}}^{{\rm{0}}}$, and horizontal-transmit and horizontal-receive, ${\sigma }_{{\rm{HH}}}^{{\rm{0}}}$) and forest AGV was found, but with significant differences exhibited between broadleaf and conifer tree-types (i.e. tree-type term has statistical significance value of *p* < 0.001 in a GLM) (Fig. 3). Equating backscatter to a set of NFI and lidar-derived vegetation structural variables, instead of AGV, adequately accounted for these differences (rendering the tree-type term non-significant *p* > 0.1). In Denmark, the combined set included mean stem size (i.e. diameter at breast height (DBH)), stem number density, mean height, lidar vegetation interception ratio (VIR) and standard deviation of heights above 1 m. VIR is a measure of the fraction of lidar pulses intercepted by canopy, defined as the ratio of number of first returns from above 1 m height to total number of first returns. In La Rioja, the combined set included mean stem size, number density, mean height, the 1^st^ percentile of heights from lidar returns above 1 m (P01) and the local terrain slope (LTS). Differences in AGV across tree-types were explained using stem number density, height and size in both study sites. The AGV-backscatter relationship also exhibited significant differences between the two sites, which were adequately accounted for in a model including all the structural properties described above (Supplementary Discussion S1 and Figure S1).

In summary, the models show that differences in backscatter across sites and tree-types can be explained by differences in macroecological forest properties of stem heights, sizes, number densities, understory and cover fraction to a highly significant extent. GLM results and residual plots are provided in Supplementary Tables S1–S6 and Figures S2–S7.

### Effect of varying structural properties on the AGV-backscatter relationship

**Figure 4:**
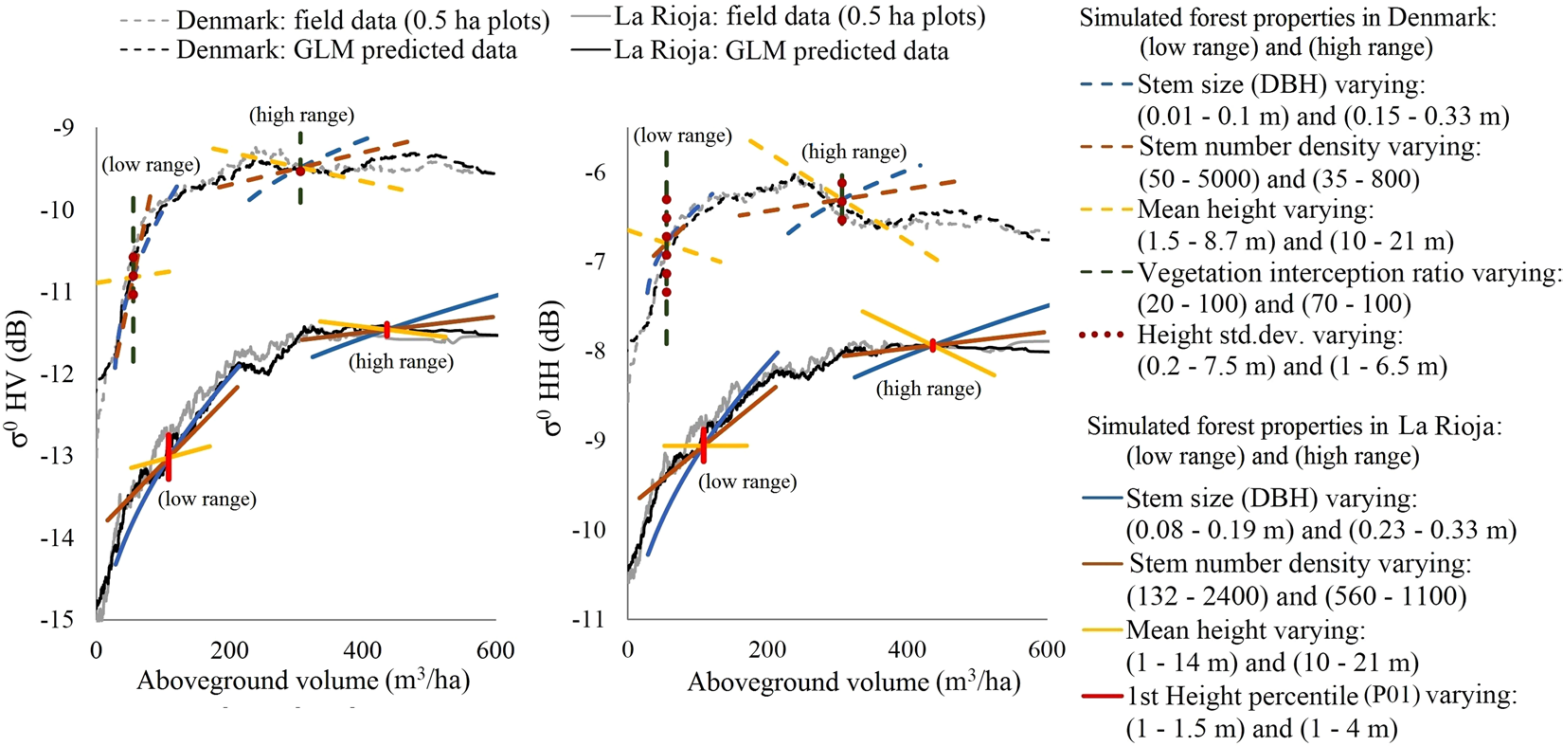
Influence of varying forest structure on the aboveground volume (AGV) and backscatter trend in simulated forests. Each structural variable is varied individually within the two sets of ranges depicted in the legend (low range and high range), causing a transition along the AGV-backscatter trend from left (low AGV values) to right (high AGV values). The general linear models (GLMs) used to predict backscatter and AGV in these simulations are provided in Supplementary Tables S1–S6. Corresponding slopes of the AGV-backscatter trends are reported in Table 1. To compare the simulations with field data and GLM predicted data, the latter are shown with smoothed moving averages of 100 observations (black and grey lines).

To study the effect of each forest structural property on the AGV-backscatter relationship, forests of different combinations of structural properties, covering two ranges (low AGV and high AGV), were simulated for La Rioja and Denmark. Each combination of structural properties was used to predict a unique AGV and backscatter value with the GLMs, and the effect of varying a single property on the predicted AGV and backscatter values was examined. Monitoring the influence of individual predictor variability on predicted AGV and backscatter using this approach revealed similar sensitivity trends in the two study sites, but different absolute values and dynamic ranges. These are demonstrated in Table 1 and Fig. 4, and summarized here:

1. Mean stem size is positively correlated to predicted backscatter in the models. A non-linear influence on the predicted AGV-backscatter relationship is observed, where the rate of increase of backscatter with increasing AGV (due to increasing stem size only) falls significantly (*p* < 0.05) as forests transition to high AGV ranges (e.g. ${\sigma }_{{\rm{HV}}}^{{\rm{0}}}$ change rate ranges 0.087–0.014 dB ha/m^3^ for AGV between 28–120 m^3^/ha in Denmark).
2. Stem number density is positively correlated to predicted backscatter in the models. A linear influence on the predicted AGV-backscatter relationship is observed, where an increase in AGV (due to increasing stem numbers only) corresponds to an increase in backscatter at a constant rate. This rate is higher in the simulated low AGV ranges than in high AGV ranges. The effect of decreasing stem number density due to natural or anthropogenic thinning on the AGV-backscatter relationship is discussed in the next section.
3. Mean stem height is positively correlated to predicted ${\sigma }_{{\rm{HV}}}^{{\rm{0}}}$ only in the simulated low AGV ranges, while negatively correlated to predicted ${\sigma }_{{\rm{HV}}}^{{\rm{0}}}$ in high AGV ranges and negatively correlated to predicted ${\sigma }_{{\rm{HH}}}^{{\rm{0}}}$ in all AGV ranges. A linear influence on the predicted AGV-backscatter relationship is observed, and the rate of decrease of ${\sigma }_{{\rm{HH}}}^{{\rm{0}}}$ with increasing AGV (due to increasing stem height only) is higher in the simulated high AGV ranges than in low AGV ranges.
4. VIR and P01 are positively correlated to predicted backscatter in the models, but show no significant contribution to predicting AGV (*p* > 0.05). Hence, the effect on the predicted AGV-backscatter relationship is a range, or scatter, of possible ${\sigma }_{{\rm{HV}}}^{{\rm{0}}}$ and ${\sigma }_{{\rm{HH}}}^{{\rm{0}}}$ values at a constant AGV value. This range is larger in the simulated low AGV ranges than in high AGV ranges.
5. The standard deviation of stem heights also shows no significant contribution to predicting AGV (*p* > 0.05). It is negatively correlated to predicted ${\sigma }_{{\rm{HV}}}^{{\rm{0}}}$ in the simulated low AGV ranges, but otherwise positively correlated to both predicted ${\sigma }_{{\rm{HV}}}^{{\rm{0}}}$ and ${\sigma }_{{\rm{HH}}}^{{\rm{0}}}$. Hence, similar to VIR and P01, the effect on the AGV-backscatter relationship is a range of possible ${\sigma }_{{\rm{HV}}}^{{\rm{0}}}$ and ${\sigma }_{{\rm{HH}}}^{{\rm{0}}}$ values at a constant AGV value.

**Table 1:** Influence of varying forest structure on the AGV-backscatter curve in simulated forests.

Structural property	Simulated ranges (D: Denmark LR: La Rioja)	Predicted AGV range	Slope of predicted AGV-${{\boldsymbol{\sigma }}}_{{\bf{HV}}}^{{\bf{0}}}$ curve (range or constant)	Slope of predicted AGV-${{\boldsymbol{\sigma }}}_{{\bf{HH}}}^{{\bf{0}}}$ curve (range or constant)
Mean stem size (i.e. mean diameter at breast height)	D: 0.01–0.1 m	28–120 m^3^/ha	0.087–0.014 dB ha/m^3^	0.044–0.007 dB ha/m^3^
	D: 0.15–0.33 m	230–418 m^3^/ha	0.006–0.003 dB ha/m^3^	0.006–0.003 dB ha/m^3^
	LR: 0.08–0.19 m	28–214 m^3^/ha	0.021–0.009 dB ha/m^3^	0.020–0.009 dB ha/m^3^
	LR: 0.23–0.33 m	325–634 m^3^/ha	0.003–0.002 dB ha/m^3^	0.003–0.002 dB ha/m^3^
Stem number density (/ha)	D: 50–5000 stems	31–81 m^3^/ha	0.039 dB ha/m^3^	0.008 dB ha/m^3^
	D: 35–800 stems	160–467 m^3^/ha	0.002 dB ha/m^3^	0.001 dB ha/m^3^
	LR: 132–2400 stems	17–212 m^3^/ha	0.009 dB ha/m^3^	0.006 dB ha/m^3^
	LR: 560–1100 stems	309–596 m^3^/ha	0.001 dB ha/m^3^	0.001 dB ha/m^3^
Mean stem height	D: 1.5–8.7 m	0.5–132 m^3^/ha	0.001 dB ha/m^3^	−0.003 dB ha/m^3^
	D: 10–21 m	176–458 m^3^/ha	−0.002 dB ha/m^3^	−0.005 dB ha/m^3^
	LR: 1–14 m	52–170 m^3^/ha	0.002 dB ha/m^3^	~0.000 dB ha/m^3^
	LR: 10–21 m	336–524 m^3^/ha	−0.001 dB ha/m^3^	−0.004 dB ha/m^3^

All other properties remain constant while mean stem size, number density and height are varied individually. Corresponding AGV-backscatter trends are shown in Fig. 4. Stem number density decreases as forests age and AGV increases, causing a decrease in backscatter at the rates specified here.

### Effect of structural development on saturation of the AGV-backscatter trend

Semi-empirical models relating AGV and backscatter in the two sites showed that the saturation of the AGV-backscatter curve (where the slope approaches 0.01 dB ha/m^3^)^32^ was exhibited at higher AGV ranges in La Rioja than Denmark. These ranges were 110–140 m^3^/ha and 90–120 m^3^/ha in La Rioja, and 70–100 m^3^/ha and 50–70 m^3^/ha in Denmark, for ${\sigma }_{{\rm{HV}}}^{{\rm{0}}}$ and ${\sigma }_{{\rm{HH}}}^{{\rm{0}}}$ respectively (Supplementary Discussion S1 and Figure S1).

**Figure 5:**
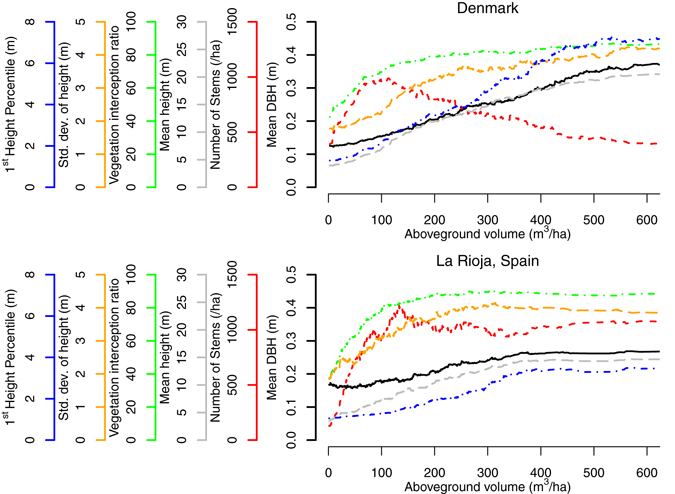
Development of various forest structural properties as aboveground volume (AGV) increases. To allow trends to be compared between the study sites, La Rioja and Denmark, only stems with diameter at breast height (DBH) > 0.075 m are included for Denmark. All data are shown as smoothed moving averages of 100 observations (ignoring missing values).

To understand the causes of the differences in saturation in the two study sites, the age-related development of the selected set of forest structural properties used to predict backscatter was examined (Fig. 5). Both Danish and La Rioja’s forests are characterized by increasing AGV with age. In Denmark, these increases correspond with rapidly decreasing stem number densities after 100 m^3^/ha, stabilizing at an average of 500 stems/ha. Increasing stem heights, DBH and P01 are recorded until 20 m, 0.35 m and 7 m on average respectively. For the same AGV values as Denmark, La Rioja’s forests have shorter and smaller-sized stems in more numerous quantities (stabilizing at height ~14 m, DBH ~0.25 m and ~1000 stems/ha on average), with P01 increasing to ~3 m only, indicative of denser understory vegetation or lower canopy components intercepting lidar pulses. From the forest simulations, it is evident that age-related changes in stem number density and mean stem height will cause a breakdown of the positive correlation between AGV and backscatter. The simulations show that as stem numbers decrease with age, a corresponding decrease in backscatter can be expected if all other structural properties are constant. Since a decrease in backscatter is not observed at low AGV ranges (Fig. 4), this stem-density induced reduction must be more than compensated for by increases in backscatter caused by other structural variables, which are also responsible for increasing AGV (e.g. stem size), resulting in a positive AGV-backscatter trend. Similarly, increasing stem heights appear to cause decreasing backscatter (except for ${\sigma }_{{\rm{HV}}}^{{\rm{0}}}$ at low AGV ranges), which must be compensated for by increases in backscatter caused by changes in other structural variables. When the rate of backscatter decrease due to decreasing stem numbers and increasing stem heights equals the rate of increase caused by other structural variables, ‘saturation’ is expected in the AGV-backscatter trend. The former rate begins to equal the latter rate at lower AGV ranges in Denmark than in La Rioja due to the regular anthropogenic thinning practices in Denmark (e.g. backscatter change due to changes in stem number and height is nearly equal to backscatter increases due to changes in stem sizes by ~50 m^3^/ha in Denmark and ~80 m^3^/ha in La Rioja). When the former rate exceeds the latter rate as forests transition to high AGV ranges, a negative correlation between AGV and backscatter can be expected, as observed in Denmark for ${\sigma }_{{\rm{HH}}}^{{\rm{0}}}$ (linear regression reveals negative slope with significance values *p* < 0.05 when AGV > 200 m^3^/ha, Supplementary Figure S1).

### The AGV-backscatter relation beyond ‘saturation’

In forests with high AGV, the prediction of AGV with SAR backscatter in empirical models becomes problematic due to the apparent saturation of the AGV-backscatter curve (for example, there is no, or a weak, correlation between backscatter and AGV at high AGV ranges (Supplementary Discussion S1)). However, equating backscatter to AGV as well as some forest structural properties revealed an increase in sensitivity of backscatter to these variables at high AGV ranges. For example, at AGV ranges beyond the saturation points, statistical models equating backscatter to AGV and VIR (i.e. linear models of the form backscatter ~AGV*VIR) consistently perform better (i.e. have higher r^2^ values and lower residual standard errors) than models that relate backscatter to AGV alone (a detailed description and comparisons of models is presented in Supplementary Discussion S2). The results serve as a demonstration of the importance of structural information in describing backscatter at high AGV ranges. They indicate that supportive information on vegetation cover fraction, for example, can allow for the prediction of AGV with backscatter using empirical models beyond saturation points.

## Discussion

The L-band backscatter relation to AGV is influenced by the age-related development of various components of forest structure (i.e. stem sizes, number densities, distribution of stem heights, and the understory and vegetation cover fraction). By empirically quantifying this influence, this study challenges the widely used explanation that the decreasing sensitivity of backscatter to AGV (i.e. signal ‘saturation’) is always a result of attenuation caused by increasing vertical canopy opacity^39^. It is found that structural changes occurring simultaneously as forests age may both increase and decrease backscatter, cancelling out and masking any differences in the SAR signal. Backscatter is *constrained* within certain bounds due to the trajectory of development of forest structure, which is governed not only by natural biophysical factors but also by various anthropogenic practices, contributing to the apparent saturation of the AGV-backscatter curve.

The results confirm the observations of a number of published empirical and theoretical studies. For example, it was found that an increase in stem sizes (DBH) resulted in an increase in backscatter when other structural properties remain constant. In the corresponding AGV-backscatter trend, backscatter was found to change at a slower rate as forests transitioned to high AGV ranges (Fig. 4); an observation seen in the theoretical modelling of scattering regime transitions as larger stems and branches transition from Rayleigh to Mie/Optical scattering^26, 27^. Similarly, at high AGV ranges, an increase in forest height resulted in a negative AGV-backscatter trend; a similar negative trend is observed in numerous empirical studies^29, 30, 40–43^. The finding is also supported by the explanation that scattering is dominated by dense upper layers of canopy rather than deeper layers, hence decreasing trunk-ground double-bounce scattering^28^, particularly ${\sigma }_{{\rm{HH}}}^{{\rm{0}}}$, and increasing signal extinction^25, 44^. In addition to the influence of vegetation cover fraction (measured as VIR), backscatter was found to be negatively correlated to understory or lower-canopy density (estimated by P01); an observation that may explain why backscatter is lower in woodlands with dense understory compared to closed forests^28^.

The influence of forest structure on the AGV-backscatter relationship raises a number of crucial discussion points for forest biomass retrieval and change detection using radar:

1. Mapping forests with SAR images may not need to be restricted to areas with low AGB/AGV, as common in previous studies^10, 12, 45^, if analyses are supplemented with adequate information on the expected forest structure, particularly maps of vegetation cover fraction or stem number densities. Although a demonstration of how forest structural information can improve the AGV-backscatter relation is provided in this study, further research on the prediction of AGV beyond the saturation point is required. It is expected that sampling schemes complementing SAR images with networks of field plots, high resolution optical data (from which stem number densities can be estimated)^46^, or lidar data, would broaden AGV retrieval ranges. Although such complementary data is expensive and may be collected at much less frequent intervals than data from SAR satellite missions^47^, even mono-temporal assessments will support accurate large-scale multi-temporal mapping using SAR. Systematic sample-based NFIs in cycles of typically either 5 or 10 years, as used in this study, are conducted in most developed countries^48^ and many developing countries. During these inventories, data on fundamental macroecological parameters are collected (e.g. basal area and stem densities) across large regions, which if extrapolated spatially using supportive environmental variables or forest-types classifications, can be highly suitable to complement SAR images for AGB/AGV estimation. Such inventories may also provide information on the differences in management practices in similar tree-type forests, which would allow establishing the range to which backscatter will be constrained and establishing AGV-backscatter trends appropriate to different structural development trajectories (e.g. different rates of stem thinning). A further opportunity for obtaining forest structural information is given by the Global Ecosystem Dynamics Investigation (GEDI) lidar, which will operate from the International Space Station and annually collect about 15 billion, cloud-free, 25 m lidar footprints^49^.
2. Although ${\sigma }_{{\rm{HV}}}^{{\rm{0}}}$ and ${\sigma }_{{\rm{HH}}}^{{\rm{0}}}$ were similarly affected by most forest structural properties, significant differences in the AGV-backscatter trends were picked out in low AGV ranges. In these ranges ${\sigma }_{{\rm{HV}}}^{{\rm{0}}}$ is positively correlated to stem heights with low standard deviations, unlike ${\sigma }_{{\rm{HH}}}^{{\rm{0}}}$ which is more sensitive to reduced ground-stem interactions with increasing height. This result suggests that polarimetry can provide complementary information that may be particularly useful in distinguishing disturbance-regrowth dynamics in young or regenerating forests, which are known to be less well quantified in comparison to deforestation^16, 50, 51^.
3. Detecting changes in forests with high ranges of AGV must be complemented with information on the types of activities leading to change, and their expected impact on forest structure. This is important since a decrease in mean height only was found to correspond to a negative AGV-backscatter trend. Hence, for example, the removal of a few tall emergent trees might lead to an increase in backscatter, and the removal of the same number of sub-canopy trees may not have as significant an impact on backscatter. Similarly, the dependence of backscatter on understory vegetation suggests that activities that selectively degrade understory may reduce forest AGV, but cause an increase in backscatter. The results suggest that our earlier study^16^ on mapping forest degradation based solely on reductions in ${\sigma }_{{\rm{HV}}}^{{\rm{0}}}$ may be inadequate. Change dynamics are indeed complicated by the dependency of forest structural components on each other, but future research must attempt to quantify the influence of various disturbances on backscatter, particularly in support of detecting subtle degradation.

Further research on the dependency of the AGV-backscatter relation on forest structure is recommended. Our results remain to be tested in vegetation that shows development outside the ranges observed in La Rioja and Denmark. For example, active management practices in these forests imply that far less structural variation is exhibited in them compared to unmanaged natural forests. Hence, although these forests provide useful test sites, the trends observed in them must be verified in other regions. Further, theoretical studies examining the impact of forest structure on the AGV-backscatter relationship have often simulated long-wavelength P-band (~70 cm) SAR, by which the effects of attenuation due to thickening forest canopy are largely eliminated^26^. Since our study uses L-band SAR, canopy cannot be completely ignored and is expected to contribute to signal saturation to some extent. Although various strata indicating canopy thickness were extracted from the lidar datasets (Supplementary Tables S7–S8), the point density was too low to examine the sole effect of attenuation on the AGV-backscatter trend. This remains an open area of research, which may be supported by the use of terrestrial lidar data from which vertical canopy density profiles may be extracted. In support, theoretical models of backscatter-vegetation interaction must also be extended to account for large scale macroecological changes in addition to increasing canopy opacity, which will provide more robust results applicable in a larger variety of forest types. Similarly, the effect of seasonal differences on lidar collected over La Rioja and Denmark, and of various other properties on backscatter (e.g. litter, vegetation moisture or stem orientation)^19, 42^, remain to be tested. Nevertheless, within the scope of this study, the models relating forest structure and SAR backscatter were robust and significant across two independent study sites, allowing the first-step towards explaining the AGV-backscatter relationship with macroecological forest properties.

In conclusion, we have shown that the inclusion of forest structural information is crucial to establishing suitable relationships between stand volume or biomass and SAR backscatter, particularly for large-scale studies across forests under different management regimes. Macroecological dynamics have essential influences on the AGV-backscatter relationship and understanding the impact of various anthropogenic disturbances on forest structure is required for accurate change detection using SAR. Future research must account for these factors in support of quantifying deforestation and forest degradation.

## Methods

### National forest inventories

Danish forests (54°34′–57°43′N and 8°04′–12°40′E) are predominantly actively managed even-aged plantations, with assisted regeneration, regular thinning and understory removal. Using manual interpretation of 0.16 m resolution aerial photography, 15 m radii NFI plots in homogeneous plantations of conifers (364 plots) and broadleaves (358 plots) were selected for analysis (Fig. 1). All trees taller than 1.3 m were measured during 2007–2011 for DBH, number density, crown cover and height, and full-tree AGV derived from tree-type specific models^52^. The topography of Denmark is flat, with a maximum elevation of ~170 m.

The forests in La Rioja province of Spain (41°55′–42°23′N and 3°07′–1°58′W) are predominantly natural, or plantations with natural regeneration, with no intensive management. Conifers (280 plots), broadleaves (575 plots) and mixed tree-types (150 plots) in plots of 25 m radii were measured for the same properties as in Denmark in 2010. Plot AGV was derived from tree-type and region-specific models for trees with DBH > 0.075 m^53, 54^. Unlike Denmark, La Rioja is mountainous, with a maximum elevation of ~2260 m and 15% of area with slopes >20° at 100 m resolution (Fig. 1).

### Airborne laser scans

In addition to the NFI measures of forest structure, 101 metrics of canopy density, height and vertical strata were extracted from discrete-return airborne lidar with point density of 0.5 pulses/m^2^ (Supplementary Tables S7–S8). The wall-to-wall data was collected over Denmark and the province of La Rioja in the leaf-off season of 2006/2007 and leaf-on season of 2010 respectively. To minimize the influence of neighbouring tree crowns at plot-edges^55, 56^ and ease comparability to coarser resolution SAR images, the extraction was done over large square-plots of 71 m × 71 m containing the circular NFI plots. Lidar metrics were compared in the square and circular plots (Supplementary Figure S8), and visual examination was conducted against aerial photography (examples in Fig. 1), to ensure that the NFI measurements were representative of the square-plots and no bias was introduced by this procedure.

### Radar images

SAR scenes (99 in Denmark and 9 in La Rioja) at processing Level 1.1^57^ were obtained from the phased array L-band SAR (PALSAR) sensor aboard ALOS over the period 2007–2010. The scenes covered the entire area of Denmark and La Rioja. Since surface moisture can affect backscatter^28, 58^, local precipitation archives^59, 60^ were examined for 5 days preceding scene acquisition to only select those taken in relatively dry weather conditions (<10 mm precipitation) and a visual examination was conducted to ensure sufficient contrast between bare-ground and vegetated areas. The scenes were converted to backscatter images, i.e. normalized radar cross-section (${\sigma }_{{\rm{HV}}}^{{\rm{0}}}$ and ${\sigma }_{{\rm{HH}}}^{{\rm{0}}}$), of 10 m pixel size. They were terrain corrected and radiometrically calibrated with 10 m resolution elevation maps derived from the airborne lidar using ASF MapReady 3.1 software (https://www.asf.alaska.edu) and the techniques described in Joshi *et al*.^16^. Backscatter was then extracted from all images overlapping each square plot (an average of 6 images per plot) and averaged, thereby minimizing speckle and requiring no additional filtering.

### Relating backscatter to forest structure

GLMs, which allow interactions between continuous and categorical predictor variables, were used as a statistical tool to examine whether the AGV-backscatter relationship differed in plots of conifers, broadleaves and mixed tree-types. The statistical models were then modified to select a set of NFI- and lidar-measured structural variables, instead of AGV, that best explained backscatter, such that the differences among tree-types were rendered non-significant (*p* > 0.05). To prevent model over-parametrization, a correlation matrix was first constructed to avoid adding highly correlated variables to the GLMs. A method of forward-selection (i.e. adding predictor variables that best explain residuals sequentially to the GLM) was then used until the most reasonable residual distributions, Akaike Information Criterion^61^ and residual deviances were achieved. Although the radar images were terrain corrected, variations of reflectivity with satellite viewing geometry can also impact backscatter^62, 63^. To ensure that the influence of topography on backscatter after terrain-correction and on vegetation structure was minimized, particularly in La Rioja, the LTS was also tested in the GLMs. LTS quantifies slope in relation to the direction of incoming SAR signals (Supplementary Methods S1) and was found to be a significant parameter in the GLMs in La Rioja.

To test how changes in forest structure affect backscatter, independent datasets of forests of different combinations of structural properties were simulated for La Rioja and Denmark. Each structural property was then varied individually to examine the sensitivity of predicted backscatter to individual predictor variability in the GLMs. To keep within realistic ranges of structural properties observed in the NFI measurements, the forests were simulated to give both low and high ranges of AGV (approximately 0–200 m^3^/ha and 200–500 m^3^/ha respectively, Table 1). Further, a single GLM that predicted AGV across tree-types was created, and the effect of varying each structural property on the predicted AGV vs. predicted SAR backscatter trend was examined.

### Relating backscatter to forest aboveground volume

A number of non-linear regression models were fitted to the AGV and backscatter datasets in the two study sites, and the models with the lowest residual standard errors and reasonable residual distribution were selected as the best-fit curves. Using the method of Watanabe *et al*.^32^, the range of AGV values at an arbitrary slope threshold of 0.01 dB ha/m^3^ was then extracted and compared between the two study regions in order to determine the rate of saturation of the AGV-backscatter curve. Further, GLMs that relate backscatter to AGV and various forest structural parameters beyond the saturation points (i.e. high AGV and backscatter ranges) were tested to demonstrate the importance of including forest structural information in the prediction of AGV with SAR backscatter. All statistics in this study were performed using R software (https://www.r-project.org/).

## Electronic supplementary material

Supplementary Information
