# Supplementary material for: Understanding ‘saturation’ of radar signals over forests
Source: Sci Rep. 2017 Jun 14;7:3505. doi: 10.1038/s41598-017-03469-3 (PMC5471195; doi:10.1038/s41598-017-03469-3)
Supplement: Supplementary file 1 — Supplementary Information [file 41598_2017_3469_MOESM1_ESM.pdf]

Supplementary Information
Understanding 'saturation' of radar signals over forests

Neha Joshi[1], Edward T. A. Mitchard[2], Matthew Brolly[3], Johannes Schumacher[1], Alfredo Fernández-Landa[4], Vivian Kvist Johannsen[1], Miguel Marchamalo[5], Rasmus Fensholt[1]

[1]Department of Geosciences and Natural Resource Management, University of Copenhagen, Copenhagen, Denmark. [2]School of GeoSciences, University of Edinburgh, Edinburgh EH9 3JN, United Kingdom. [3]School of Environment and Technology, University of Brighton, Cockcroft Building, Lewes Road, Brighton BN2 4GJ, UK. [4]AGRESTA Sociedad Cooperativa, Soria, Spain. [5]Departamento de Ingeniería y Morfología del Terreno, Universidad Politécnica de Madrid, E-28040 Madrid, Spain. Correspondence and requests for materials should be addressed to N.J. (neha.joshi@gisat.cz).

Summary of Supplementary Information

This supplementary material to the main text contains additional information on the methods, including equations and general linear models (GLMs), used in the manuscript. Further, it provides details of the airborne lidar scans collected over Denmark and La Rioja, Spain. All general linear models and statistics were conducted using R software (`https://www.r-project.org/`) and the inbuilt packages *nlstools*, *ggplot2*, *rlm*, *zoo* and *Hmisc*. Lidar data was processed using FUSION software (`http://forsys.cfr.washington.edu/fusion/fusionlatest.html`).

Supplementary Methods S1: The local terrain slope (LTS) is a map that measures the slope and aspect of the ground surface with respect to the satellite, calculated as:

$$\mathbf{LTS} = \arctan(\tan(\mathbf{slope}) - \cos(\mathbf{aspect} - a)) \tag{1}$$

where a is the look angle of the satellite relative to north used to correct the aspect, such that 0° is facing the satellite. In the case of polar orbiting SAR satellites such as ALOS, the value of a is normally 90° or 270°. We used the ALOS inclination angle of 97.9°.

LTS is expected to be proportional to the radiometric signal caused by local slope in radar imagery. To ensure that a single value of LTS was extracted for each National Forest Inventory (NFI) plot, the lidar-derived DEMs were resampled by averaging to 70 m × 70 m pixel size before mapping slope, aspect, and extracting an LTS value for each plot.

Supplementary Discussion S1: The AGV-backscatter relationship exhibited significant differences in the two study locations, La Rioja and Denmark (p<0.001 in a GLM with a categorical variable for study site). These differences were explained by a set of forest structural variables instead of AGV. This set included mean stem size (i.e. DBH), stem number density, mean height, lidar vegetation interception ratio (VIR), standard deviation of heights above 1 m, the 1st percentile of heights from lidar returns above 1 m (P01) and the local terrain slope (LTS), which rendered the categorical variable for study sites non-significant (p>0.1).

In order to determine the rate at which backscatter (i.e. the normalized radar cross-section, σ^0_{HV} and σ^0_{HH}) saturates to increasing AGV, a number of non-linear regression models were fitted to the AGV and backscatter datasets. The simplified form of the Water-Could Model was found to most accurately predict backscatter (lowest Akaike Information Criterion, lowest residual deviances and well distributed residuals) in both locations (equation and plot provided in Supplementary Figure S1). The equations are undefined where backscatter is equal to or greater than the coefficients $a+c$ dB (here, the models are undefined where σ^0_{HV} and σ^0_{HH} exceed -9.5 dB and -6.4 dB respectively in Denmark, and -11.4 dB and -7.9 dB respectively in La Rioja province). Using the method of *Watanabe et al.*[1], the range of AGV values at an arbitrary slope threshold of 0.01 dB/(m^3/ha) (i.e. change in backscatter occurring from a unit change in forest volume) was extracted and compared between the two study regions. It was found that the saturation of the AGV-backscatter curve (where the slope approaches 0.01 dB/(m^3/ha)) was exhibited at higher AGV values in La Rioja than Denmark, i.e. between 110–140 m^3/ha (σ^0_{HV}) and 90–120 m^3/ha (σ^0_{HH}) in La Rioja, and 70–100 m^3/ha (σ^0_{HV}) and 50–70 m^3/ha (σ^0_{HH}) in Denmark.

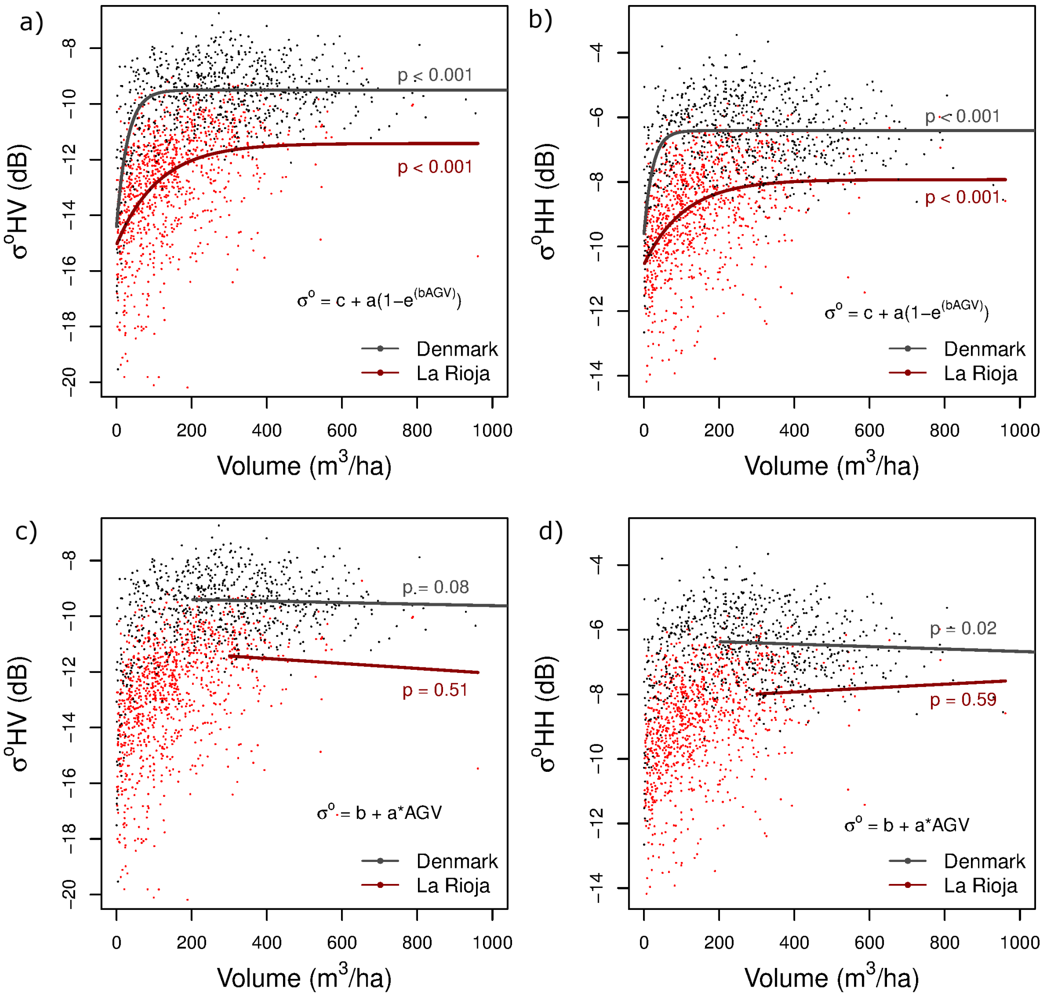

Supplementary Figure S1: (a) and (b) L-band SAR backscatter and aboveground forest stand volume (AGV) data from Denmark's and La Rioja's national forest inventory. A significant difference in the relation between AGV and backscatter is observed over the two sites (p<0.001). Further, data is fitted using a simplified form of the Water-Could Model (equation and p-value for all coefficients in the model is provided on the plot). (c) and (d) Backscatter and AGV fitted using a linear model where AGV>200 m^3/ha in Denmark and AGV>300 m^3/ha in La Rioja to demonstrate the relation between the variables at high AGV values (equation and p-value for slope is provided on the plot). A significant negative trend between backscatter and AGV is observed in Denmark, particularly for σ^0_{HH}.

Supplementary Discussion S2: At high ranges the relation between backscatter and AGV breaks down, and the prediction of AGV becomes problematic (as discussed in Supplementary Discussion S1). In order to test whether additional information on forest structure can improve the AGV-backscatter relation, several GLMs (or equivalently, linear models) of the form [backscatter $\sim$ AGV * forest structural properties] were constructed in the two study sites. Across both study sites, it was found that adding information on the vegetation cover fraction (here, lidar–derived VIR) in the models improves the relation (i.e. provides higher r^2 values and lower residual standard errors). Models including forest structure consistently perform better than those that equate backscatter to AGV alone. Most improvement was observed for σ^0_{HV} in Denmark, which has a non-significant correlation (p>0.05) to AGV, but a significant correlation to AGV and VIR (p<0.01 for all coefficients and $r^2 = 0.20$), when AGV> 85 m^3/ha (results are demonstrated below).

Linear model relating σ^0_{HV} to AGV (where AGV > 85 m^3/ha) in Denmark:

```
Call:
lm(formula = LogHV ~ AGV)

Residuals:
Min      1Q  Median      3Q     Max
-3.6375 -0.7124  0.0504  0.7013  2.7716

Coefficients:
Estimate   Std. Error   t value    Pr(>|t|)
(Intercept)   -9.49503280  0.06705413  -141.603   <2e-16 ***
AGV           -0.00005952  0.00015081    -0.395    0.693
---
Signif. codes:  0 *** 0.001 ** 0.01 * 0.05 . 0.1   1

Residual standard error: 1.023 on 604 degrees of freedom
Multiple R-squared:  0.0002578,Adjusted R-squared:  -0.001397
F-statistic: 0.1558 on 1 and 604 DF,  p-value: 0.6932
```

Linear model relating AGV (where AGV > 85 m^3/ha) to VIR in Denmark:

```
Call:
lm(formula = AGV ~ VIR)

Residuals:
Min     1Q Median     3Q    Max
-312.0 -129.6  -40.7   74.1 3934.4

Coefficients:
Estimate    Std. Error  t value   Pr(>|t|)
(Intercept)   81.5264    66.8866    1.219     0.223
VIR            3.3095     0.8163    4.054     0.0000568 ***
---
Signif. codes:  0 *** 0.001 ** 0.01 * 0.05 . 0.1   1

Residual standard error: 272.3 on 604 degrees of freedom
Multiple R-squared:  0.02649,Adjusted R-squared:  0.02488
F-statistic: 16.44 on 1 and 604 DF,  p-value: 0.00005684
```

Linear model relating σ^0_{HV} to AGV (where AGV $>$ 85 m^3/ha) and VIR in Denmark:

```
Call:
lm(formula = LogHV ~ AGV * VIR)

Residuals:
Min       1Q   Median       3Q      Max
-2.88941 -0.60436  0.05542  0.64256  2.30868

Coefficients:
              Estimate     Std. Error   t value   Pr(>|t|)
(Intercept) -13.07533228   0.37297754   -35.057  < 2e-16 ***
AGV           0.00308864   0.00106191     2.909  0.00376 **
VIR           0.04547252   0.00461517     9.853  < 2e-16 ***
AGV:VIR      -0.00004141   0.00001277    -3.244  0.00124 **
---
Signif. codes:  0 *** 0.001 ** 0.01 * 0.05 . 0.1   1

Residual standard error: 0.9128 on 602 degrees of freedom
Multiple R-squared:  0.2065,Adjusted R-squared:  0.2026
F-statistic: 52.23 on 3 and 602 DF,  p-value: < 2.2e-16
```

Supplementary Table S1: General linear model relating σ^0_{HV} to forest structural variables in Denmark. The variables that best described σ^0_{HV} (LogHV) included stem size (MeanDBH), stem number (N), stem height (MeanHeight), vegetation interception ratio (VIR) and the standard deviation of stem heights (HeightSDEV). Corresponding residual plots are provided in Supplementary Figure S2. Adding a categorical variable of tree-types (broadleaves or conifers) renders interaction terms of the model non-significant (p<0.1), implying that forest structural variables sufficiently explain differences in backscatter over different tree-types.

```
Call: glm(formula = LogHV ~ MeanDBH * N * MeanHeight * VIR * HeightSDEV)

Deviance Residuals:
Min       1Q   Median       3Q      Max
-4.0532  -0.5787   0.0743   0.6003   3.6314

Coefficients:                                                 Estimate     Std. Error    t value  Pr(>|t|)
(Intercept)                                                -13.9976915214  0.6524404932 -21.454  < 2e-16 ***
MeanDBH                                                    -14.5954348743  6.5317726971  -2.235 0.025768 *
N                                                           -0.0017999156  0.0003012106  -5.976 3.67e-09 ***
MeanHeight                                                   0.1905727940  0.1877424067   1.015 0.310425
VIR                                                          0.0642150598  0.0131437166   4.886 1.28e-06 ***
HeightSDEV                                                  -0.1495774399  0.3191292958  -0.469 0.639429
MeanDBH:N                                                    0.0494952243  0.0076915448   6.435 2.31e-10 ***
MeanDBH:MeanHeight                                           1.6633515302  0.6399518449   2.599 0.009544 **
N:MeanHeight                                                 0.0001987059  0.0000471736   4.212 2.86e-05 ***
MeanDBH:VIR                                                  0.1598150973  0.0986050920   1.621 0.105526
N:VIR                                                        0.0000164863  0.0000048179   3.422 0.000659 ***
MeanHeight:VIR                                              -0.0036435914  0.0022976701  -1.586 0.113248
MeanDBH:HeightSDEV                                           2.6475692762  1.9819715123   1.336 0.182047
N:HeightSDEV                                                 0.0005796392  0.0001458529   3.974 7.81e-05 ***
MeanHeight:HeightSDEV                                       -0.0142187260  0.0338778072  -0.420 0.674831
VIR:HeightSDEV                                              -0.0081896735  0.0057421950  -1.426 0.154255
MeanDBH:N:MeanHeight                                        -0.0064031628  0.0013263571  -4.828 1.70e-06 ***
MeanDBH:N:VIR                                               -0.0005538429  0.0001039558  -5.328 1.35e-07 ***
MeanDBH:MeanHeight:VIR                                      -0.0174944555  0.0076157494  -2.297 0.021909 *
N:MeanHeight:VIR                                            -0.0000022691  0.0000006492  -3.495 0.000504 ***
MeanDBH:N:HeightSDEV                                        -0.0108202694  0.0037239878  -2.906 0.003783 **
MeanDBH:MeanHeight:HeightSDEV                               -0.3067644984  0.1513138439  -2.027 0.043012 *
N:MeanHeight:HeightSDEV                                     -0.0000514175  0.0000104272  -4.931 1.03e-06 ***
MeanDBH:VIR:HeightSDEV                                      -0.0040034098  0.0296595701  -0.135 0.892668
N:VIR:HeightSDEV                                            -0.0000064347  0.0000018597  -3.460 0.000573 ***
MeanHeight:VIR:HeightSDEV                                    0.0007952104  0.0004587274   1.734 0.083451 .
MeanDBH:N:MeanHeight:VIR                                     0.0000775971  0.0000155898   4.977 8.15e-07 ***
MeanDBH:N:MeanHeight:HeightSDEV                              0.0015013579  0.0003207224   4.681 3.43e-06 ***
MeanDBH:N:VIR:HeightSDEV                                     0.0001586197  0.0000482317   3.289 0.001058 **
MeanDBH:MeanHeight:VIR:HeightSDEV                            0.0020728233  0.0018928050   1.095 0.273852
N:MeanHeight:VIR:HeightSDEV                                  0.0000006221  0.0000001460   4.260 2.33e-05 ***
MeanDBH:N:MeanHeight:VIR:HeightSDEV                         -0.0000200952  0.0000039808  -5.048 5.72e-07 ***
---
Signif. codes:  0 *** 0.001 ** 0.01 * 0.05 . 0.1   1

(Dispersion parameter for gaussian family taken to be 0.93906)

Null deviance: 1608.10  on 721  degrees of freedom
Residual deviance:  647.95  on 690  degrees of freedom
AIC: 2036.8

Number of Fisher Scoring iterations: 2

Equivalent linear model provides R2 = 0.5971 and residual standard error = 0.9691 dB.
```

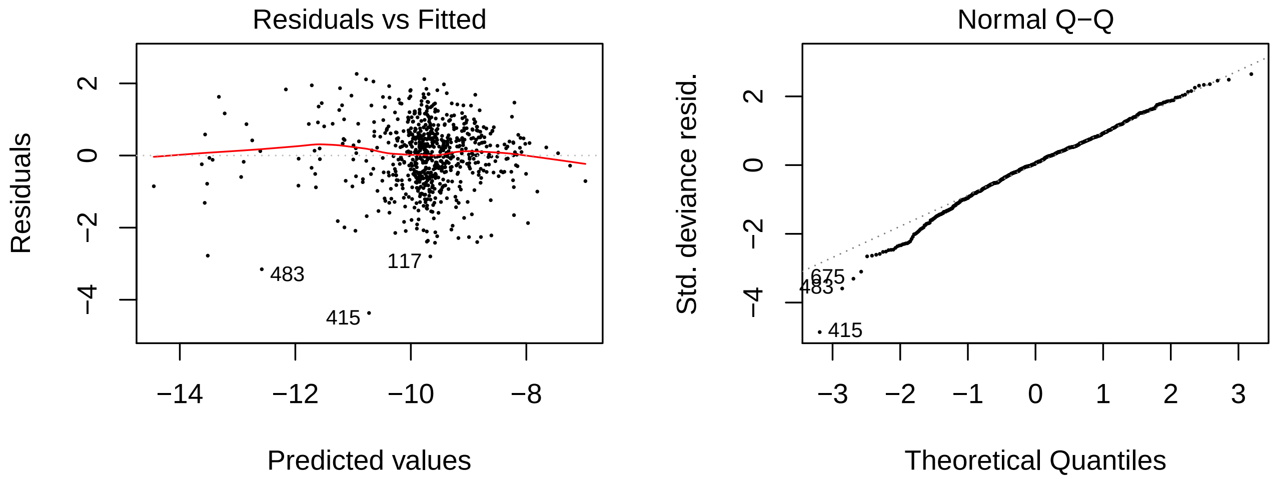

Supplementary Figure S2: Residual plots for general linear model relating σ^0_{HV} (in dB scale) to forest structural variables in Denmark (Supplementary Table S1).

Supplementary Table S2: General linear model relating σ^0_{HH} to forest structural variables in Denmark. The variables that best described σ^0_{HH} (LogHH) included stem size (MeanDBH),stem number (N), stem height (MeanHeight), vegetation interception ratio (VIR) and the standard deviation of stem heights (HeightSDEV). Corresponding residual plots are provided in Supplementary Figure S3. Similar to the model in Supplementary Table S1, adding a tree-types term to the model renders it non-significant (p<0.1).

```
Call: glm(formula = LogHH ~ MeanDBH * N * MeanHeight * VIR * HeightSDEV)

Deviance Residuals:
Min       1Q   Median       3Q      Max
-3.4292  -0.4757   0.0526   0.5907   4.0988

Coefficients:                                                Estimate       Std. Error    t value   Pr(>|t|)
(Intercept)                                              -9.0985788735   0.6031120577 -15.086      < 2e-16 ***
MeanDBH                                                 -19.9376382024   6.0379312945  -3.302     0.001009 **
N                                                        -0.0011886070   0.0002784373  -4.269 0.0000223990 ***
MeanHeight                                                0.2467685872   0.1735479486   1.422     0.155506
VIR                                                       0.0289135131   0.0121499724   2.380     0.017597 *
HeightSDEV                                                0.0098406911   0.2950011967   0.033     0.973399
MeanDBH:N                                                 0.0391649551   0.0071100177   5.508 0.0000000511 ***
MeanDBH:MeanHeight                                        1.7761151078   0.5915676265   3.002     0.002775 **
N:MeanHeight                                              0.0001182462   0.0000436070   2.712     0.006862 **
MeanDBH:VIR                                               0.3475324756   0.0911499524   3.813     0.000150 ***
N:VIR                                                     0.0000106470   0.0000044537   2.391     0.017087 *
MeanHeight:VIR                                           -0.0040595272   0.0021239524  -1.911     0.056379 .
MeanDBH:HeightSDEV                                        3.9339713165   1.8321225147   2.147     0.032123 *
N:HeightSDEV                                              0.0002976175   0.0001348256   2.207     0.027613 *
MeanHeight:HeightSDEV                                    -0.0439749617   0.0313164407  -1.404     0.160705
VIR:HeightSDEV                                            0.0014833905   0.0053080505   0.279     0.779975
MeanDBH:N:MeanHeight                                     -0.0058040663   0.0012260765  -4.734 0.0000026741 ***
MeanDBH:N:VIR                                            -0.0004367809   0.0000960961  -4.545 0.0000064785 ***
MeanDBH:MeanHeight:VIR                                   -0.0244310271   0.0070399528  -3.470     0.000552 ***
N:MeanHeight:VIR                                         -0.0000012959   0.0000006001  -2.159     0.031171 *
MeanDBH:N:HeightSDEV                                     -0.0087486244   0.0034424319  -2.541     0.011258 *
MeanDBH:MeanHeight:HeightSDEV                            -0.3578657712   0.1398736049  -2.558     0.010725 *
N:MeanHeight:HeightSDEV                                  -0.0000289467   0.0000096389  -3.003     0.002769 **
MeanDBH:VIR:HeightSDEV                                   -0.0579325067   0.0274171277  -2.113     0.034959 *
N:VIR:HeightSDEV                                         -0.0000032478   0.0000017191  -1.889     0.059272 .
MeanHeight:VIR:HeightSDEV                                 0.0005949094   0.0004240449   1.403     0.161085
MeanDBH:N:MeanHeight:VIR                                  0.0000693700   0.0000144111   4.814 0.0000018220 ***
MeanDBH:N:MeanHeight:HeightSDEV                           0.0013701453   0.0002964739   4.621 0.0000045477 ***
MeanDBH:N:VIR:HeightSDEV                                  0.0001110736   0.0000445851   2.491     0.012963 *
MeanDBH:MeanHeight:VIR:HeightSDEV                         0.0045843235   0.0017496975   2.620     0.008985 **
N:MeanHeight:VIR:HeightSDEV                               0.0000003477   0.0000001350   2.575     0.010220 *
MeanDBH:N:MeanHeight:VIR:HeightSDEV                      -0.0000169173   0.0000036798  -4.597 0.0000050902 ***
---
Signif. codes:  0 *** 0.001 ** 0.01 * 0.05 . 0.1   1

(Dispersion parameter for gaussian family taken to be 0.8024307)

Null deviance: 1094.07  on 721  degrees of freedom
Residual deviance:  553.68  on 690  degrees of freedom
AIC: 1923.3

Number of Fisher Scoring iterations: 2

Equivalent linear model provides R2 = 0.4939 and residual standard error = 0.8958 dB.
```

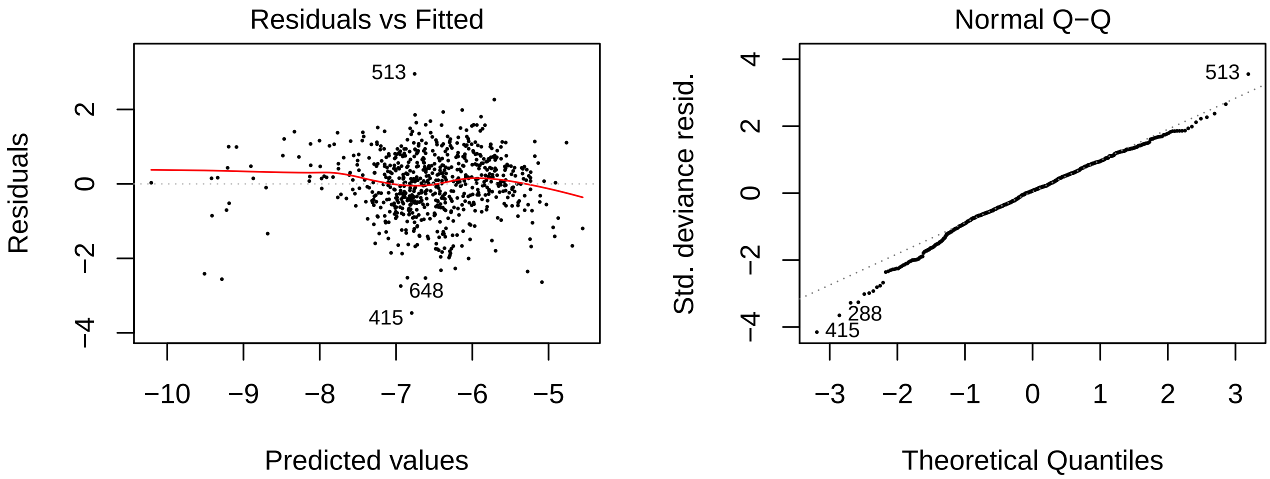

Supplementary Figure S3: Residual plots for general linear model relating σ^0_{HH} (in dB scale) to forest structural variables in Denmark (Supplementary Table S2).

Supplementary Table S3: General linear model relating σ^0_{HV} to forest structural variables in La Rioja. The variables that best described σ^0_{HV} (LogHV) included stem size (MeanDBH), stem number (N), stem height (MeanHeight), local terrain slope (LTS) and the 1st percentile of heights from lidar returns above 1 m (P01). Corresponding residual plots are provided in Supplementary Figure S4. Adding a categorical variable of tree-types (broadleaves, conifers or mixed) renders interaction terms of the model non-significant (p<0.1), implying that forest structural variables and LTS sufficiently explain differences in backscatter over different tree-types.

```
Call: glm(formula = LogHV ~ MeanDBH * N * MeanHeight * LTS * P01)

Deviance Residuals:
Min       1Q   Median       3Q      Max
-7.1999  -0.8022   0.0574   0.9073   3.6573

Coefficients:                           Estimate     Std. Error  t value Pr(>|t|)
(Intercept)                         -18.381269383   1.125455242 -16.332  < 2e-16 ***
MeanDBH                              -1.533137352   3.922259707  -0.391 0.695971
N                                    -0.004159778   0.001074357  -3.872 0.000115 ***
MeanHeight                            0.135553352   0.099423025   1.363 0.173072
LTS                                   0.180996603   0.101311199   1.787 0.074323 .
P01                                   4.367352397   0.977846213   4.466 8.89e-06 ***
MeanDBH:N                             0.046858571   0.006624485   7.074 2.88e-12 ***
MeanDBH:MeanHeight                    0.925195521   0.323964229   2.856 0.004383 **
N:MeanHeight                          0.000315461   0.000103976   3.034 0.002477 **
MeanDBH:LTS                           0.414666748   0.348367668   1.190 0.234214
N:LTS                                 0.000249099   0.000093637   2.660 0.007936 **
MeanHeight:LTS                       -0.000028312   0.007905562  -0.004 0.997143
MeanDBH:P01                          -6.485450272   3.086281283  -2.101 0.035864 *
N:P01                                 0.000737177   0.000777584   0.948 0.343347
MeanHeight:P01                       -0.196089613   0.065989865  -2.972 0.003036 **
LTS:P01                              -0.126244589   0.091775702  -1.376 0.169269
MeanDBH:N:MeanHeight                 -0.002859340   0.000576872  -4.957 8.46e-07 ***
MeanDBH:N:LTS                        -0.001944388   0.000565222  -3.440 0.000606 ***
MeanDBH:MeanHeight:LTS               -0.065437825   0.027091992  -2.415 0.015902 *
N:MeanHeight:LTS                     -0.000022423   0.000007847  -2.858 0.004358 **
MeanDBH:N:P01                        -0.013548350   0.004452783  -3.043 0.002408 **
MeanDBH:MeanHeight:P01                0.188666596   0.182620776   1.033 0.301811
N:MeanHeight:P01                     -0.000089064   0.000059455  -1.498 0.134454
MeanDBH:LTS:P01                      -0.030959150   0.297772736  -0.104 0.917215
N:LTS:P01                            -0.000166519   0.000076636  -2.173 0.030032 *
MeanHeight:LTS:P01                    0.005914272   0.006103716   0.969 0.332805
MeanDBH:N:MeanHeight:LTS              0.000169689   0.000046435   3.654 0.000272 ***
MeanDBH:N:MeanHeight:P01              0.000937630   0.000308001   3.044 0.002395 **
MeanDBH:N:LTS:P01                     0.001287516   0.000435654   2.955 0.003198 **
MeanDBH:MeanHeight:LTS:P01            0.010221503   0.019344226   0.528 0.597342
N:MeanHeight:LTS:P01                  0.000012537   0.000005491   2.283 0.022621 *
MeanDBH:N:MeanHeight:LTS:P01         -0.000091921   0.000030461  -3.018 0.002614 **
---
Signif. codes:  0 *** 0.001 ** 0.01 * 0.05 . 0.1   1

(Dispersion parameter for gaussian family taken to be 1.848623)
Null deviance: 4221.8  on 1004  degrees of freedom
Residual deviance: 1798.7  on  973  degrees of freedom
AIC: 3503.1
Number of Fisher Scoring iterations: 2

Equivalent linear model provides R2 = 0.574 and residual standard error = 1.36 dB.
```

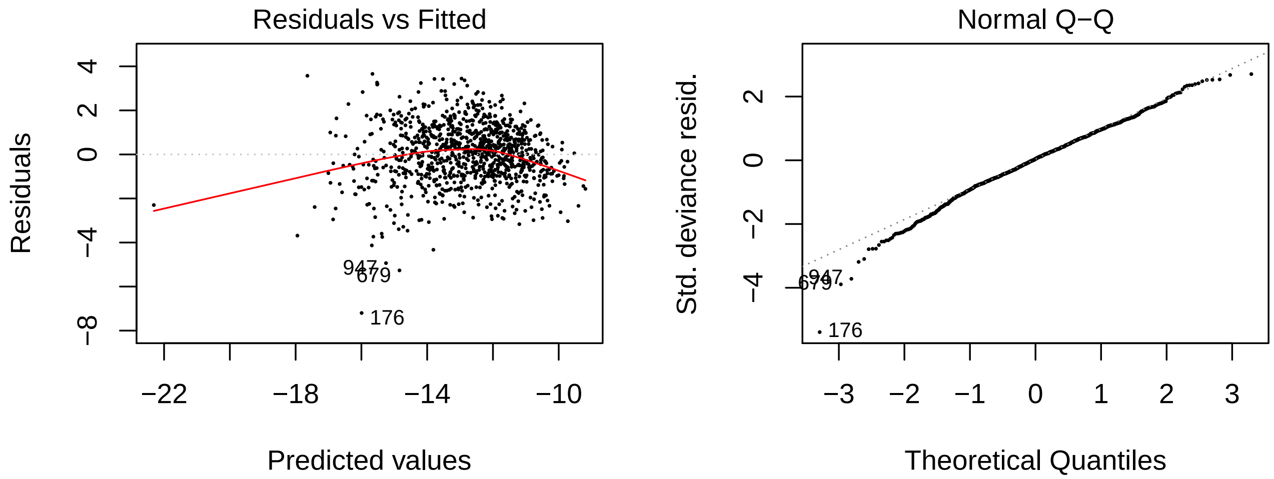

Supplementary Figure S4: Residual plots for general linear model relating σ^0_{HV} (in dB scale) to forest structural variables in La Rioja (Supplementary Table S3).

Supplementary Table S4: General linear model relating σ^0_{HH} to forest structural variables in La Rioja. The variables that best described σ^0_{HH} (LogHH) included stem size (MeanDBH), stem number (N), stem height (MeanHeight), , local terrain slope (LTS) and the 1st percentile of heights from lidar returns above 1 m (P01). Corresponding residual plots are provided in Supplementary Figure S5. Similar to the model in Supplementary Table S3, adding a tree-types term to the model renders it non-significant (p<0.1).

```
Call: glm(formula = LogHH ~ MeanDBH * N * MeanHeight * LTS * P01)

Deviance Residuals:
Min       1Q   Median       3Q      Max
-6.7760  -0.7502   0.0462   0.7819   3.0895

Coefficients:                            Estimate    Std. Error   t value Pr(>|t|)
(Intercept)                          -12.820046796   1.003572416 -12.774  < 2e-16 ***
MeanDBH                               -1.835752533   3.497492842  -0.525 0.599788
N                                     -0.003904003   0.000958008  -4.075 4.97e-05 ***
MeanHeight                             0.152517919   0.088655863   1.720 0.085689 .
LTS                                   -0.028652291   0.090339554  -0.317 0.751189
P01                                    2.572006466   0.871948923   2.950 0.003257 **
MeanDBH:N                              0.040221330   0.005907077   6.809 1.72e-11 ***
MeanDBH:MeanHeight                     0.544616056   0.288880048   1.885 0.059692 .
N:MeanHeight                           0.000268403   0.000092715   2.895 0.003877 **
MeanDBH:LTS                            0.582218884   0.310640681   1.874 0.061195 .
N:LTS                                  0.000182594   0.000083496   2.187 0.028991 *
MeanHeight:LTS                         0.011804659   0.007049418   1.675 0.094342 .
MeanDBH:P01                           -2.126281253   2.752047927  -0.773 0.439936
N:P01                                  0.001103730   0.000693375   1.592 0.111749
MeanHeight:P01                        -0.130639023   0.058843396  -2.220 0.026641 *
LTS:P01                                0.035990412   0.081836717   0.440 0.660192
MeanDBH:N:MeanHeight                  -0.002476935   0.000514399  -4.815 1.70e-06 ***
MeanDBH:N:LTS                         -0.000840308   0.000504011  -1.667 0.095788 .
MeanDBH:MeanHeight:LTS                -0.067623138   0.024158025  -2.799 0.005224 **
N:MeanHeight:LTS                      -0.000019029   0.000006997  -2.720 0.006652 **
MeanDBH:N:P01                         -0.012005309   0.003970562  -3.024 0.002564 **
MeanDBH:MeanHeight:P01                 0.036672414   0.162843592   0.225 0.821871
N:MeanHeight:P01                      -0.000117120   0.000053016  -2.209 0.027397 *
MeanDBH:LTS:P01                       -0.341193486   0.265525001  -1.285 0.199106
N:LTS:P01                             -0.000154762   0.000068337  -2.265 0.023751 *
MeanHeight:LTS:P01                    -0.004673976   0.005442705  -0.859 0.390685
MeanDBH:N:MeanHeight:LTS               0.000090412   0.000041406   2.184 0.029235 *
MeanDBH:N:MeanHeight:P01               0.000920376   0.000274645   3.351 0.000836 ***
MeanDBH:N:LTS:P01                      0.000839046   0.000388474   2.160 0.031028 *
MeanDBH:MeanHeight:LTS:P01             0.030264625   0.017249315   1.755 0.079653 .
N:MeanHeight:LTS:P01                   0.000012512   0.000004896   2.556 0.010752 *
MeanDBH:N:MeanHeight:LTS:P01          -0.000064365   0.000027162  -2.370 0.017999 *
---
Signif. codes:  0 *** 0.001 ** 0.01 * 0.05 . 0.1   1

(Dispersion parameter for gaussian family taken to be 1.469905)

Null deviance: 2698.9  on 1004  degrees of freedom
Residual deviance: 1430.2  on  973  degrees of freedom
AIC: 3272.7

Number of Fisher Scoring iterations: 2

Equivalent linear model provides R2 = 0.4701 and residual standard error = 1.212 dB.
```

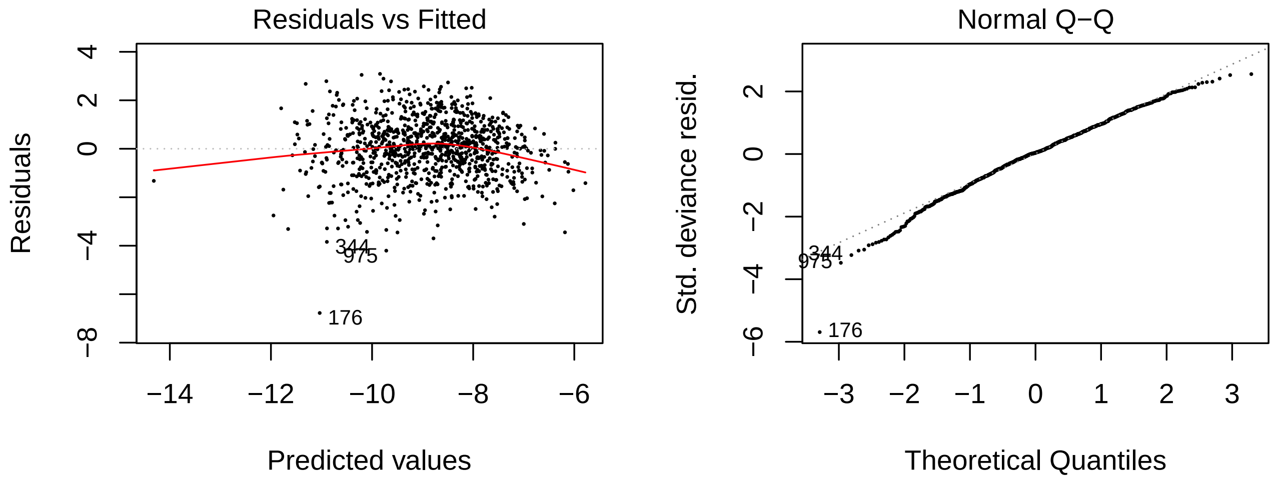

Supplementary Figure S5: Residual plots for general linear model relating σ^0_{HH} (in dB scale) to forest structural variables in La Rioja (Supplementary Table S4).

Supplementary Table S5: General linear model relating aboveground volume (AGV) to forest structural variables in Denmark. The variables that best described AGV included stem size squared (MeanDBHsq), stem number (N) and stem height (MeanHeight). Corresponding residual plots are provided in Supplementary Figure S6. Since residuals plots suggest the model may under-predict AGV at very high ranges (i.e.>500 m^3/ha), these ranges are not covered in the simulations that use predicted AGV values in this study (see Table 1 in main text).

```
Call:glm(formula = AGV ~ MeanDBHsq * N * MeanHeight)

Deviance Residuals:
Min      1Q  Median      3Q     Max
-403.4   -52.9   -17.4    17.9  3239.2
Coefficients:                                   Estimate     Std. Error  t value    Pr(>|t|)
(Intercept)                                  -25.0872399    33.7690988  -0.743      0.4578
MeanDBHsq                                  -2387.7457161   529.5526254  -4.509 0.000007612 ***
N                                             -0.0083484     0.0085899  -0.972      0.3314
MeanHeight                                    13.5189400     2.4372289   5.547 0.000000041 ***
MeanDBHsq:N                                    3.4792744     1.4736565   2.361      0.0185 *
MeanDBHsq:MeanHeight                         122.7922816    23.2303162   5.286 0.000000166 ***
N:MeanHeight                                   0.0010885     0.0005623   1.936      0.0533 .
MeanDBHsq:N:MeanHeight                         0.2228925     0.1055036   2.113      0.0350 *
---
Signif. codes:  0 *** 0.001 ** 0.01 * 0.05 . 0.1   1

(Dispersion parameter for gaussian family taken to be 39782.98)
Null deviance: 55632841  on 721  degrees of freedom
Residual deviance: 28405050  on 714  degrees of freedom
AIC: 9705.7
Number of Fisher Scoring iterations: 2
Equivalent linear model provides R2 = 0.4894 and residual standard error = 199.5 m3/ha.
```

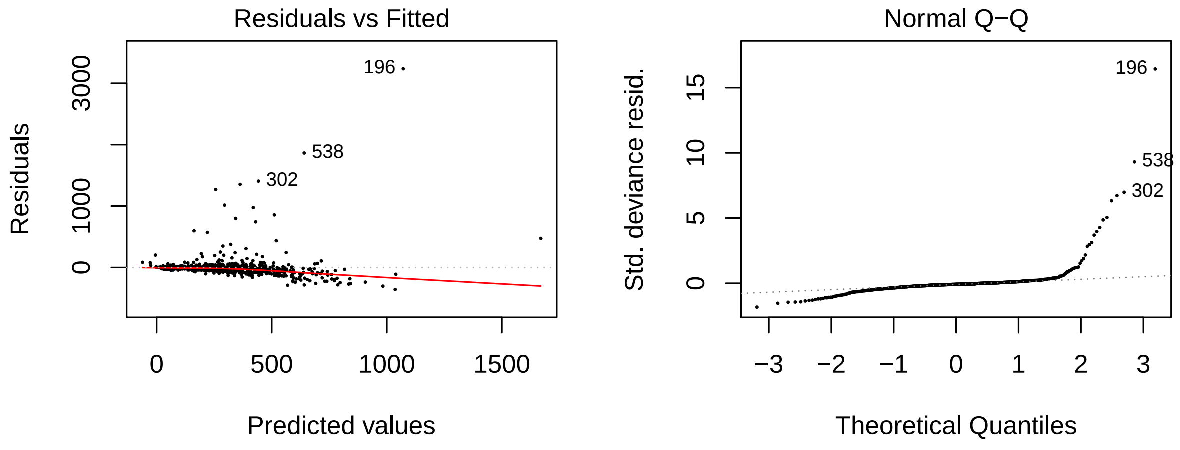

Supplementary Figure S6: Residual plots for general linear model relating aboveground volume (AGV in units m^3/ha) to forest structural variables in Denmark (Supplementary Table S5).

Supplementary Table S6: General linear model relating aboveground volume (AGV) to forest structural variables in La Rioja. The variables that best described AGV included stem size squared (MeanDBHsq), stem number (N) and stem height (MeanHeight). Corresponding residual plots are provided in Supplementary Figure S7. Since residuals plots suggest the model may under-predict AGV at very high ranges (i.e.>650 m^3/ha), these ranges are not covered in the simulations that use predicted AGV values in this study (see Table 1 in main text).

```
Call:glm(formula = AGV ~ MeanDBHsq * N * MeanHeight)

Deviance Residuals:
Min       1Q   Median       3Q      Max
-185.73   -14.24    -2.13    11.57   329.63
Coefficients:                    Estimate  Std. Error  t value Pr(>|t|)
(Intercept)                    -7.2454667   5.5566242  -1.304 0.192557
MeanDBHsq                      -2.3882687  67.3870230  -0.035 0.971735
N                              -0.0211878   0.0057629  -3.677 0.000249 ***
MeanHeight                      2.0304608   0.7500378   2.707 0.006902 **
MeanDBHsq:N                     3.3182102   0.2159797  15.364  < 2e-16 ***
MeanDBHsq:MeanHeight          -10.7204200   6.8151596  -1.573 0.116030
N:MeanHeight                    0.0016519   0.0006862   2.407 0.016251 *
MeanDBHsq:N:MeanHeight          0.2426034   0.0194892  12.448  < 2e-16 ***
---
Signif. codes:  0 *** 0.001 ** 0.01 * 0.05 . 0.1   1

(Dispersion parameter for gaussian family taken to be 1110.265)
Null deviance: 14994185  on 1004  degrees of freedom
Residual deviance:  1106935  on  997  degrees of freedom
AIC: 9909.5
Number of Fisher Scoring iterations: 2
Equivalent linear model provides R2 = 0.9262 and residual standard error = 33.32 m3/ha.
```

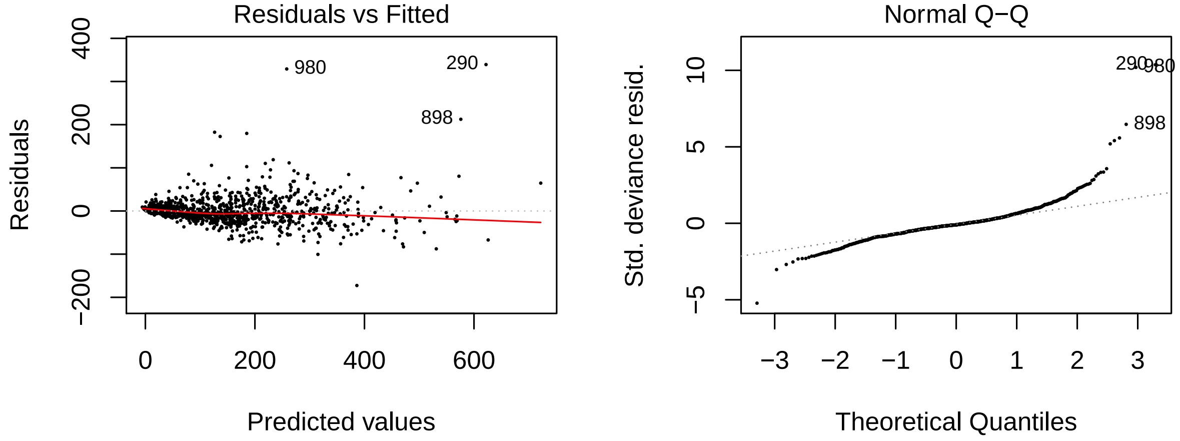

Supplementary Figure S7: Residual plots for general linear model relating aboveground volume (AGV in units m^3/ha) to forest structural variables in La Rioja (Supplementary Table S6).

Supplementary Table S7: Summary of the airborne lidar and flight data collected over Denmark in spring 2006 and fall 2006/spring 2007, and over La Rioja, Spain, in summer 2010.

	Denmark	La Rioja, Spain
Date	Fall 2006/2007	September 2010
System	Optech ALTM 3100	LEICA ALS50-II
Flying altitude	1600 m	2165 m
Pulse repetition frequency	70 kHz	150 kHz
Scan angle	$\pm 24°$	$\pm 25°$
Average point density	0.5 pulse/m^2	0.5 pulse/m^2
Average footprint size	50 cm	150 cm
Estimated horizontal accuracy	80 cm	40 cm
Estimated vertical accuracy	10 cm	20 cm

Supplementary Table S8: List of lidar metrics extracted from the lidar point cloud data collected over Denmark and La Rioja. Where height thresholds are required, metrics were extracted and tested for two specified height values, above-ground returns from >1 m height and >2 m height.

Total number of returns
Count of returns by return number
Minimum
Maximum
Mean
Median (output as 50th percentile)
Mode
Standard deviation
Variance
Coefficient of variation
Interquartile distance
Skewness
Kurtosis
AAD (Average Absolute Deviation)
MADMedian (Median of the absolute deviations from the overall median)
MADMode (Median of the absolute deviations from the overall mode)
L-moments (L1, L2, L3, L4)
L-moment skewness
L-moment kurtosis
Percentile values (1st, 5th, 10th ... 90th, 95th, 99th percentiles)
Canopy relief ratio ((mean - min) / (max min))
Generalized means for the 2nd and 3rd power (Elev quadratic mean and Elev cubic mean)
Percentage of first returns above a specified height (canopy cover estimate)
Percentage of first returns above the mean height/elevation
Percentage of first returns above the mode height/elevation
Percentage of all returns above a specified height
Percentage of all returns above the mean height/elevation
Percentage of all returns above the mode height/elevation
Number of returns above a specified height / total first returns * 100
Number of returns above the mean height / total first returns * 100
Number of returns above the mode height / total first returns * 100
Percentage of returns from vertical space between ground and max. height divided into 10 strata

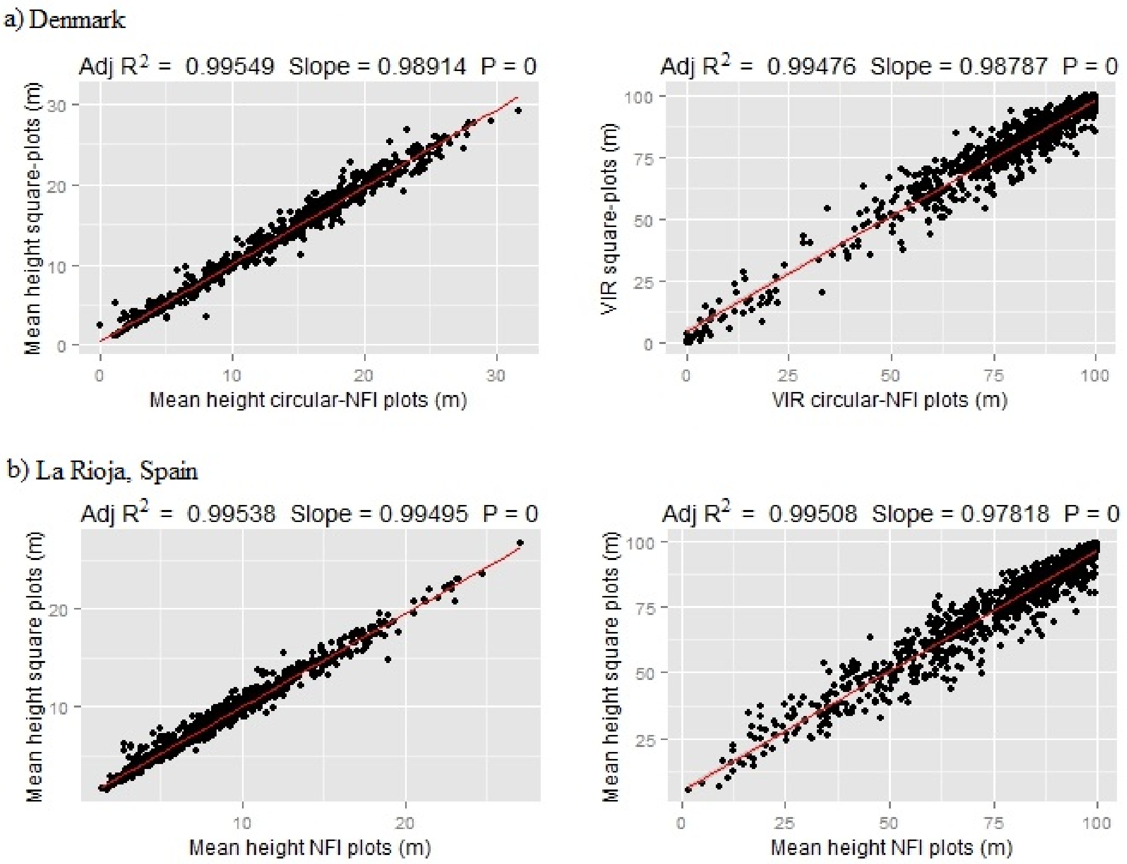

Supplementary Figure S8: Examples of comparisons of lidar metrics (mean forest height and vegetation interception ratio, VIR) collected over circular National Forest Inventory plots and larger square plots (71 m × 71 m size) in Denmark (a) and La Rioja, Spain (b).

References

[1] Watanabe, M., M. Shimada, A. Rosenqvist, T. Tadono, M. Matsuoka, S. A. Romshoo, K. Ohta, R. Furuta, K. Nakamura, and T. Moriyama (2006), Forest structure dependency of the relation between L-Band sigma-naught and biophysical parameters, *Geoscience and Remote Sensing, IEEE Transactions on*, *44*(11), 3154–3165, doi:10.1109/TGRS.2006.880632.
